# Tuning the Shell Elasticity of Phospholipid-Coated Microbubbles via Palmitic Acid Doping

**DOI:** 10.1021/acs.langmuir.5c02618

**Published:** 2025-10-18

**Authors:** Benjamin van Elburg, Kim Bruil, Guillaume Lajoinie, Mark Borden, Michel Versluis, Tim Segers

**Affiliations:** † Physics of Fluids Group, Max Planck Center Twente for Complex Fluid Dynamics, Faculty of Science and Technology, 3230 University of Twente, Enschede 7500 AE, The Netherlands; ‡ Department of Mechanical Engineering, 1877 University of Colorado, Boulder, Colorado 80309-0427, United States; ¶ BIOS/Lab on a Chip Group, Max Planck Center Twente for Complex Fluid Dynamics, MESA+ Institute for Nanotechnology, Faculty of Electrical Engineering, Mathematics and Computer Science, 3230 University of Twente, Enschede 7500 AE, The Netherlands

## Abstract

Phospholipid-coated microbubbles have been developed as blood pool agents for contrast-enhanced ultrasound imaging. Tuning their acoustic response is key to expanding their use beyond contrast imaging alone. Here, we demonstrate that the shell elasticity of coated microbubbles can be controlled over 1 order of magnitude, from 0.5 N/m up to 4.5 N/m, by doping the native shell with palmitic acid. Characterization of shell elasticity as a function of bubble surface area via ambient pressure-controlled acoustic attenuation measurements revealed that the increased shell elasticity is confined to a narrow region around the equilibrium bubble surface area. Upon expansion of just 1–2% in bubble surface area, the surface elasticity rapidly drops to levels observed in non-PA-doped bubbles. The results further demonstrated that shell viscosity also varies with bubble surface area, which may further enhance nonlinear bubble dynamics. Dilatational surface tension curves, obtained by numerically integrating the elasticity curves, were used as input to a nonlinear bubble dynamics model based on a Rayleigh–Plesset-type equation. The results demonstrate that the controlled shell elasticity offered by this work allows microbubbles to be tuned in nonlinear acoustic response, significantly enhancing the sensitivity of their subharmonic response for a range of applications, including noninvasive pressure sensing.

## Graphical abstract

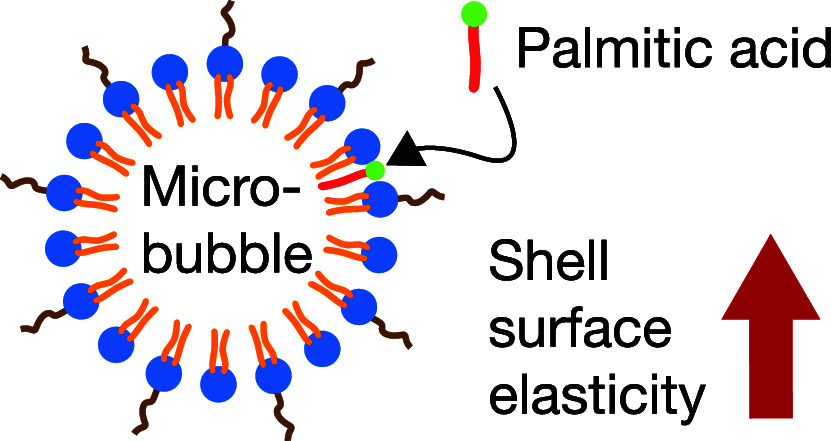

## Introduction

Phospholipid-coated microbubbles have been used as ultrasound contrast agents (UCAs) for nearly three decades.^1^ Precise control over the acoustic response of these microbubbles is essential for expanding their applications beyond blood pool visualization.^1^ UCAs are suspensions of shell-stabilized microbubbles, typically 1–10 μm in diameter, whose acoustic behavior is governed by resonance. The resonance frequency is inversely proportional to bubble size,^2^ but it is also strongly influenced by the viscoelastic properties of the lipid shell. An increase in shell elasticity raises the resonance frequency, while an increase in dilatational shell viscosity increases damping. Shell elasticity χ = *A*dσ/d*A* arises from surface tension gradients (dσ/d*A*) that can be induced by acoustically driven variations in bubble surface area d*A*. For a lipid-coated bubble, the shell elasticity is highly nonlinear because it can vanish upon both shell buckling and rupture.^3–5^ These variations in shell elasticity with surface area (∼ ∂^2^σ/∂*A* ^2^) have been shown to enhance nonlinear bubble dynamics,^6^ producing strong echoes containing harmonics of the driving frequency even at low acoustic driving pressures (<40 kPa).^7,8^ Today, these harmonics are exploited in perfusion imaging to distinguish bubble echoes from linear tissue echoes.^9^ However, the harmonic content is highly sensitive, not only to resonance and bubble shell properties, but also to ambient pressure,^10–14^ medium acidity,^15^ and surface interactions such as ligand-mediated binding.^16–18^ Thus, achieving precise control over both bubble size and dilatational shell elasticity may unlock rational bubble design for new applications,^19,20^ including blood pressure sensing and “multicolor” ultrasound molecular imaging.^21^

Size control can be achieved through microfluidic bubble formation in a flow-focusing device.^22–24^ Here, a gas thread is focused between a liquid flow through a constriction where it pinches off to release uniformly sized bubbles at a production rate that can exceed 1 million bubbles per second. By carefully selecting lipid and gas mixture compositions,^25,26^ optimizing bubble production temperature,^27^ and refining collection and processing methods,^27,28^ bubble functionality can be achieved, and bubble stability can be maintained even under physiological ambient pressure variations^29^ and in vivo.^30^ While these techniques enable precise control over bubble size and stability, achieving control over surface elasticity and its gradients—crucial for tuning nonlinear bubble dynamics—remains elusive.

A broad range of surface elasticity values has been reported in the literature, spanning from nearly zero to 3.5 N/m.^1,3,4,18,19,31–43^ However, the relation between shell composition and surface elasticity remains unclear. Interestingly, in the field of artificial pulmonary surfactants, the addition of palmitic acid (PA), a single acyl chain fatty acid (C16:0), to a DPPC monolayer—one of the primary components of pulmonary surfactant^44^—has been shown to increase surface elasticity at low-frequency surface dilatation rates corresponding to physiological breathing conditions.^45,46^ Inspired by these findings, as well as by the presence of PA in the clinically available UCAs Sonovue and Levovist,^1,47^ this study explores the role of PA in tuning the dilatational surface elasticity of monodisperse lipid-coated microbubbles oscillating at the microsecond time scale, with the goal of achieving control over surface elasticity, its gradient, and the resulting nonlinear bubble dynamics.

Shell elasticity χ is typically characterized via high-speed imaging or acoustic attenuation spectroscopy, both conducted at atmospheric pressure. These approaches provide a single effective χ value, providing no insight into its variation during ultrasound-driven microbubble oscillations. However, obtaining the full dilatational surface elasticity curve (χ­(*A*)) is essential for accurately predicting the harmonic echo response.^6,48,49^ Recently, Segers et al.^4^ demonstrated that χ­(*A*) can be obtained from repeated narrowband ultrasound attenuation measurements at very low acoustic driving pressures (<5 kPa) on a monodisperse microbubble suspension, while the microbubble surface area *A* is varied by changing the ambient pressure at a seconds time scale. The shell elasticity is then acoustically probed by attenuation spectroscopy at MHz-frequency bubble oscillations. By fitting a linearized bubble dynamics model—a Rayleigh–Plesset-type equation incorporating shell viscoelasticity^3^—to each attenuation curve, χ­(*A*) can can be extracted and subsequently numerically integrated to obtain σ­(*A*). In the present work, these ambient pressure-dependent acoustic attenuation measurements are used to investigate the role of palmitic acid doping on the surface elasticity of the microbubble shell. First, the shell elasticity is characterized at atmospheric pressure for 36 monodisperse microbubble suspensions with varying molar fractions of PA. Second, for four PA concentrations, the full χ­(*A*) curves are measured.

## Materials and Methods

### Shell Compositions

The formulations contained the phospholipid DSPC, the PEGylated lipid DPPE-PEG5000 and the fatty acid palmitic acid (PA) in the following molar fractions:

$$\frac{9}{10}\left(1-\phi_{\mathrm{PA}}\right):\frac{1}{10}\left(1-\phi_{\mathrm{PA}}\right):\phi_{\mathrm{PA}}$$

Thus, the molar ratio of DSPC to DPPE-PEG5000 was kept constant at 9:1. The molar fraction of PA (ϕ_*PA*_) was varied between 0 and 0.8 for a total of 8 concentrations. The total concentration of DSPC and DPPE was always 12.5 mg/mL while PA was added to further increase the total lipid concentration. The phospholipid dispersions were prepared as before.^25^ In short, the lipids were dissolved in a 2:1 v/v mixture of chloroform and methanol. After a 10 min period of mixing at 70 °C, the solvents were evaporated using a rotavapor for at least 6 h. The dried mixture was rehydrated in Isoton (Beckman Coulter, ISOTON II Diluent) at 70 °C for 5 min. The resulting dispersion was sonicated for 5 min using a tip sonicator (Branson Sonifier, model 250) at an output power of 30% until the liquid became translucent. Sonication disrupts large lipid aggregates into smaller ( O ­(100 nm)^25,27^ ) liposomes, increasing liposome concentration, which in turn reduces on-chip bubble coalescence as described by Segers et al.,^25,27^ and prevents clogging of the micron-sized fluidic structures.

### Microfluidic Monodisperse Microbubble Production

Phospholipid-coated monodisperse microbubble suspensions were produced in the flow-focusing device described in,^4^ and shown in Figure 1A. Microfluidically formed lipid-coated bubbles are inherently unstable until their size has diffusively reduced typically by a factor of 1.5–3, depending on the lipid formulation.^25^ The size reduction mechanically compresses the monolayer shell until a surface pressure is reached that balances the surface tension of the surrounding medium.^26^ To ensure rapid bubble stabilization and prevent foam formation due to Ostwald ripening,^50^ the microbubble filling gas entering the flow-focusing device comprised a mixture of C_4_F_10_ and CO_2_ at a ratio of 15:85 by volume. Additionally, to minimize bubble coalescence in the outlet of the flow-focusing device, the device was kept at 60 °C.^27^ The bubbles were collected at room temperature in a sealed glass vial prefilled with C_4_F_10_ gas (Figure 1A), allowing bubble stabilization in a C_4_F_10_ environment. During bubble collection, a venting needle was inserted into the vial to maintain atmospheric pressure. The stabilized bubbles contain nearly pure C_4_F_10_ gas as demonstrated by Segers et al.^50^

**Figure 1:**
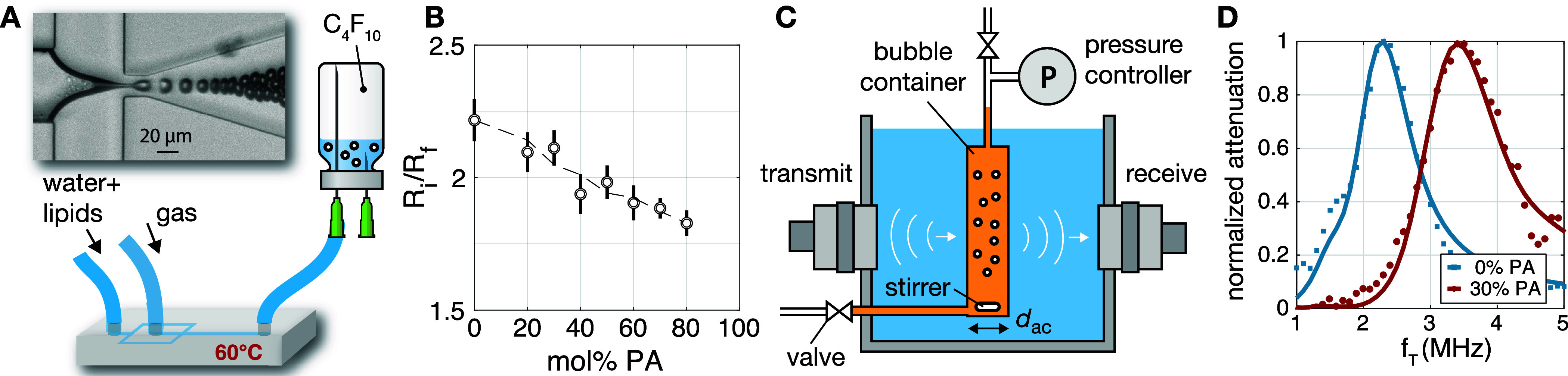
(A) Monodisperse phospholipid-coated microbubbles were formed at a temperature of 60 °C in a flow-focusing device and collected in an airtight glass vial prefilled with perfluorobutane (C_4_F_10_) gas. (B) Ratio of the initial bubble radius *R*_*i*_ after pinch off in the flow-focusing device to the final stable bubble radius *R*_*f*_ in the collection vial. The vertical lines indicate the standard deviation. (C) Microbubbles were characterized by ambient pressure-controlled acoustic attenuation spectroscopy. Narrowband ultrasound pulses were transmitted through a stirred bubble suspension confined in a pressure-controlled sample holder positioned in the focal region of the confocally aligned transmit and receive transducers. (D) Two typical attenuation curves as a function of transmit frequency *f*_*T*_ for two bubble suspensions with an equal modal bubble radius of 2.4 μm but with different palmitic acid molar fractions of 0 and 30 mol %.

**Figure 2:**
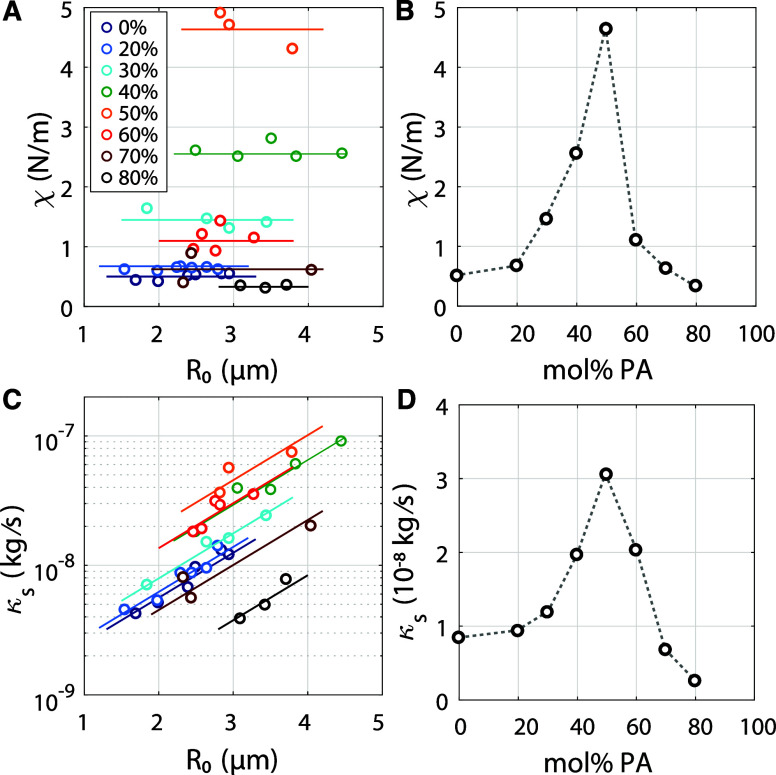
(A) Surface elasticity as a function of equilibrium microbubble radius (circles). The solid lines represent the average χ per PA concentration. (B) Surface elasticity as a function of molar percentage of palmitic acid showing that surface elasticity can be tuned from 0.5 up to 4.5 N/m at 50 mol % palmitic acid, while above 50%, the surface elasticity drops. (C) Shell viscosity as a function of microbubble radius. Shell viscosity exponentially increases with bubble radius. The solid lines show exponential fits to the data: κ_*s*_ = *c*_1_ · *e* ^*c*_2_ *R*_0_^, with *c*_2_ = 8.45× 10^5^, and where *c*_1_ varied with PA concentration. (D) Shell viscosity is calculated using the exponential fits for a 2.5-μm radius bubble. The curve follows a similar trend as in (B) with a maximum at 50 mol % PA.

**Figure 3:**
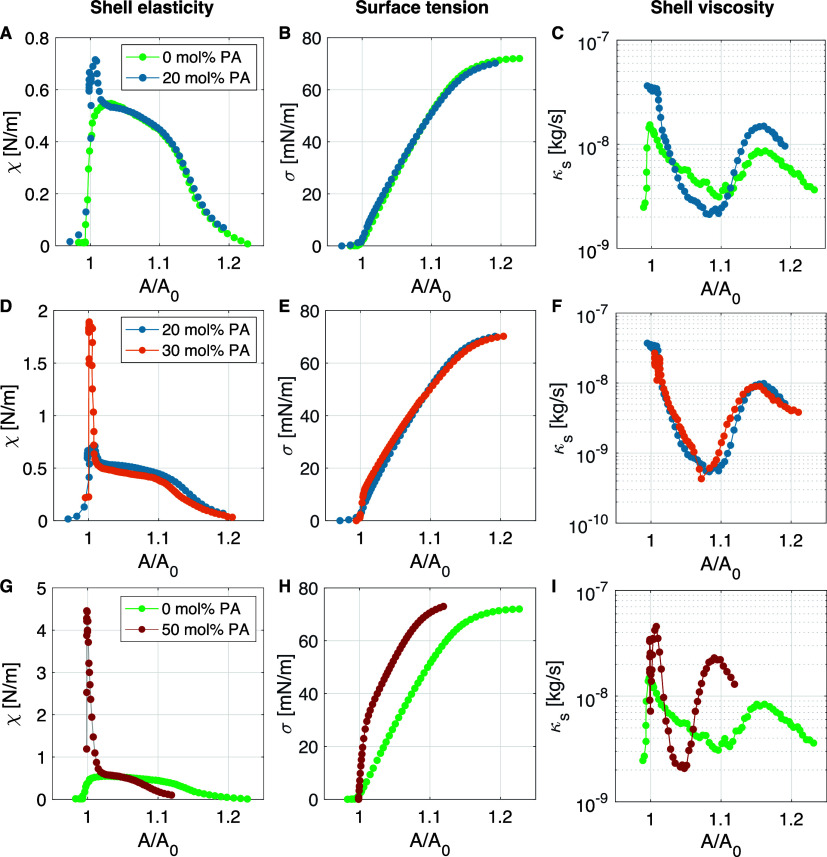
(A) Dilatational shell elasticity measured for bubbles coated with DSPC mixed with DPPE-PEG5000 at a 9:1 molar ratio (gray data points), compared to those coated with the same mixture but doped with 20 mol % palmitic acid (PA, blue data points). (B) Corresponding surface tension curves and (C) simultaneously obtained dilatational shell viscosity curves. (D–F) Shell elasticity, surface tension, and shell viscosity curves for bubbles doped with 20 and 30 mol % PA. (G–I) Shell elasticity, surface tension, and shell viscosity curves for bubbles without PA, compared to those doped with 50 mol % PA.

**Figure 4:**
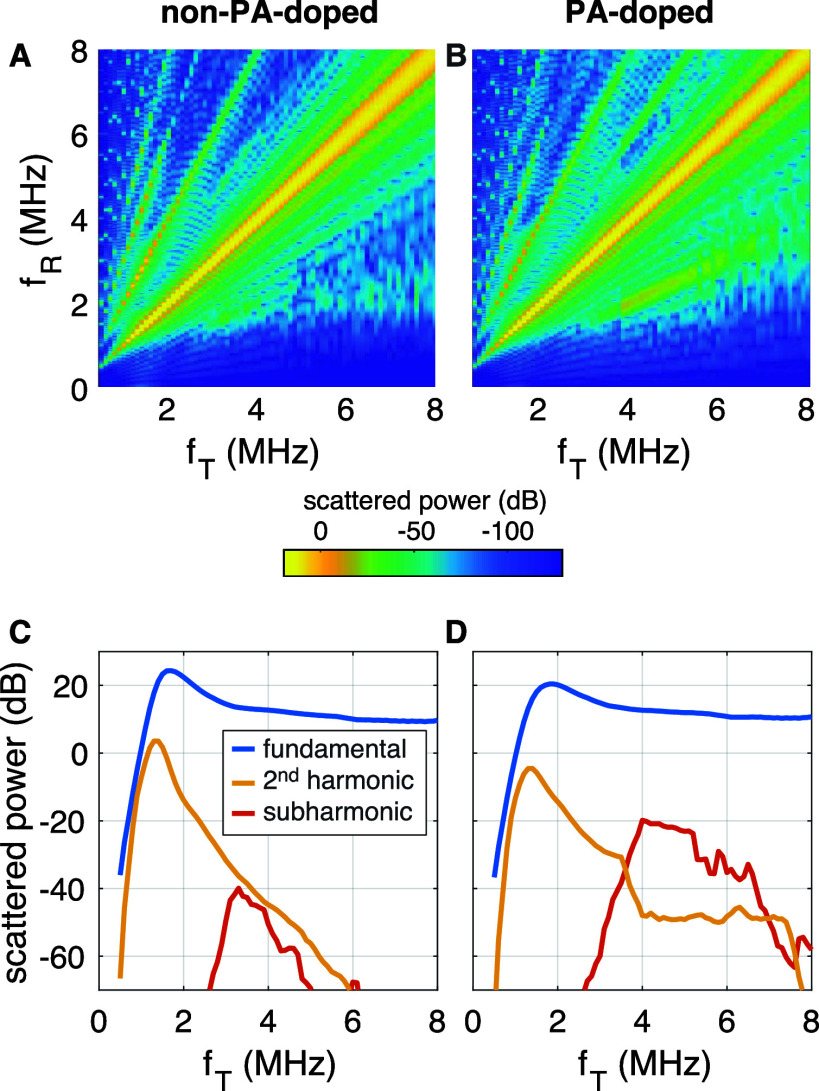
Simulated scattered power received at a distance of 2.54 cm from the bubble at frequency *f*_*R*_ as a function of acoustic driving frequency *f*_*T*_ for a 2.5-μm radius bubble. (A) Simulation result for a bubble not doped with PA and (B) for that doped with 50 mol % PA. The acoustic driving pulse had a length of 16-cycles and an acoustic pressure amplitude of 75 kPa. The surface tension curves of Figure 3H were input to the modeling. (C) and (D) show the scattered power at the fundamental, second harmonic, and subharmonic corresponding to the spectral maps in (A) and (B), respectively.

The size reduction of microbubbles with varying molar fractions of PA after formation was quantified using optical ultrahigh-speed imaging of the flow-focusing nozzle at 10 million frames per second (Shimadzu HPV-X2). The obtained images were processed as described in^37^ to obtain the initial bubble radius *R*_*i*_. After bubble collection, the final stable bubble radius *R*_*f*_ was measured using a Coulter Counter (Multisizer 3, 30 μm aperture tube, Beckman Coulter, Brea, CA, USA). Prior to Coulter Counter measurements, the bubbles were diluted 2000 times in air-saturated Isoton and allowed to equilibrate via gas exchange with their new environment for 3 min.^51^ All size distributions are shown in Figure 5 in the Appendix. The ratio of initial to final stable bubble radius is plotted in Figure 1B. For each PA concentration, this ratio is based on measurements from at least three and up to eight independent bubble suspensions formed using the same lipid formulation where the error bars in Figure 1B represent the standard deviation at each PA concentration. The plot shows that bubbles shrink less during stabilization as the molar fraction of PA increases. Therefore, to produce bubble suspensions of equal final size with different PA concentrations, the initial bubble size must be adjusted accordingly.

**A1 fig5:**
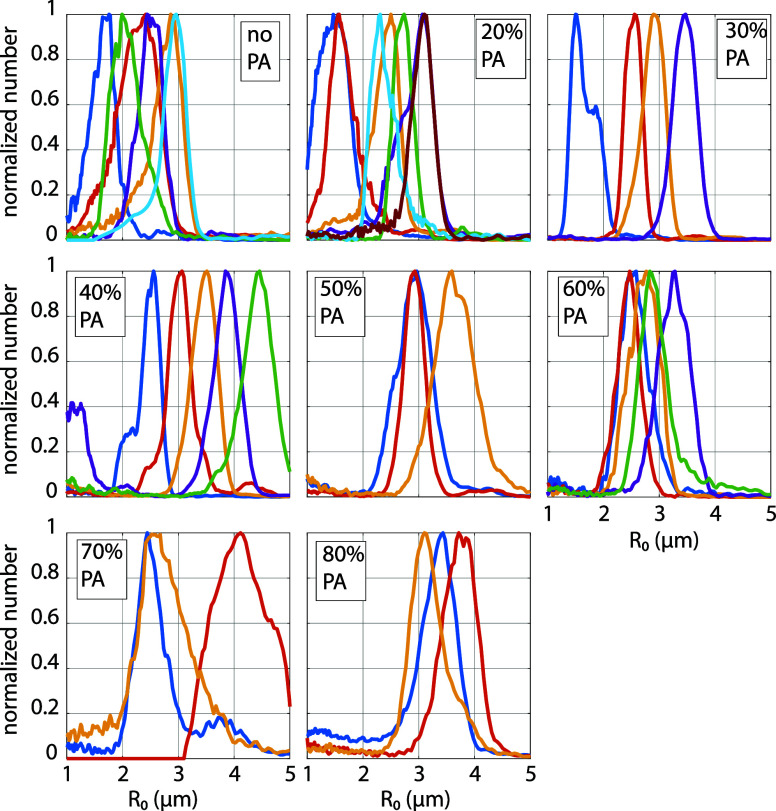
Size distributions of all employed microbubble suspensions, measured using a Coulter Counter.

### Acoustic Measurement of the Dilatational Shell Elasticity

The setup, experimental procedure, and data analysis method followed exactly the detailed description by Segers et al.^4^ In short, attenuation spectra were obtained by transmitting narrowband 30 cycle sinusoidal ultrasound pulses through a microbubble suspension, diluted 2 × 10^4^ times in air-saturated Isoton and confined in an ambient pressure-controlled sample holder (Figure 1C). A transmit transducer (4.78 cm focal length, 2.25 MHz center frequency, V304, Panametrics) generated the ultrasound pulses, while a receiving transducer (4.93 cm focal length, 5 MHz center frequency, V307, Panametrics) was confocally aligned to record the attenuated signals. The transmit transducer was pressure calibrated using a 0.2 mm needle hydrophone such that its transmit acoustic pressure was 2.5 kPa for all transmitted frequencies. Attenuation at the transmit frequency *f*_*T*_ was then calculated as follows:^52^

$$\alpha \left(f_{T}\right)=-\frac{10}{d}\log_{10}\;\frac{|V_{\mathrm{bub}}\left(f_{t}\right){|}^{2}}{|V_{\mathrm{ref}}\left(f_{t}\right){|}^{2}}$$ 1

where |*V*_bub_(*f*_*t*_)|^2^ is the power of the bubble signal around *f*_*T*_ and |*V*_ref_(*f*_*T*_)|^2^ is the power of a reference signal measured for a sample holder filled with Isoton without bubbles. The transmit pulse frequency *f*_*T*_ was swept from 0.5 to 5.0 MHz in 100 kHz increments. Every 100 ms over a 5 s period, a new attenuation spectrum was measured, with each individual attenuation spectrum acquisition taking 46 ms. During this period, the ambient pressure in the sample container was gradually increased from atmospheric pressure to 40 kPa overpressure. A second experiment was conducted on a fresh bubble sample from the same vial, where the ambient pressure was deceased from atmospheric pressure to 35 kPa below atmospheric pressure. The pressure in the bubble sample holder was monitored by a pressure sensor (SMC, PSE533M5) connected to a digital oscilloscope (Picoscope 5444d).

The bubble radius as a function of ambient pressure was obtained using optical microscopy imaging, where the bubbles were confined in a microchannel (IBIDI slide), and subjected to the same pressurization and depressurization conditions as during the acoustic attenuation spectroscopy measurements. From the recorded images, the bubble size was obtained from the inflection point of the angle-averaged intensity profiles of single bubbles, as described in detail in.^37^

After data collection, the attenuation curves and corresponding bubble size distributions *n*(*R*) were used to obtain χ­(*A*) by fitting a modeled attenuation spectrum to each measured attenuation spectrum. Again, all details can be found in.^4^ In brief, we use the Rayleigh–Plesset-type equation given by Marmottant et al.^3^ as a starting point:

$$\rho \left(\ddot{R}R+\frac{3}{2}{\dot{R}}^{2}\right)=\left(P_{0}+\frac{2\sigma (R_{0})}{R_{0}}\right){\left(\frac{R_{0}}{R}\right)}^{3\kappa }\left(1-\frac{3\kappa \dot{R}}{c}\right)-P_{0}-P_{A}(t)-\frac{2\sigma (R)}{R}-\frac{4\mu \dot{R}}{R}-\frac{4\kappa_{s}\dot{R}}{R^{2}}$$ 2

where ρ is the liquid density, μ the liquid viscosity, *c* the speed of sound in the liquid, κ the polytropic exponent of the gas inside the bubble, with *P*_0_ the ambient pressure and *P*_A_ the acoustic driving pressure. *R*_0_ is the initial bubble radius, *R* the time-dependent radius of the bubble and the overdots denote its time derivatives. κ_*s*_ accounts for the dilatational viscosity of the shell. The effective surface tension is captured by σ­(*R*).

Eq 2 can be linearized to describe bubble dynamics at small amplitudes of oscillation around the equilibrium radius *R*_0_. For linear microbubble oscillations, the angular eigenfrequency is given by^53^

$$\omega_{0}=\frac{1}{R_{0}}\sqrt{\frac{1}{\rho }\left(3\kappa P_{\mathrm{amb}}+\left(3\kappa -1\right)\frac{2\sigma \left(R_{0}\right)}{R_{0}}+\frac{4\chi }{R_{0}}\right)}$$ 3

where *P*_amb_ is the ambient pressure and σ­(*R*_0_) the surface tension of the bubble interface at bubble radius *R*_0_.

Energy dissipation in bubble oscillator system is characterized by the dimensionless damping coefficients:

$$\delta_{\mathrm{rad}}=\frac{\omega R_{0}}{c_{L}},\delta_{\mathrm{vis}}=\frac{4\mu_{L}}{\rho \omega_{0}R_{0}^{2}},\delta_{\mathrm{shell}}=\frac{4\kappa_{s}}{\rho \omega_{0}R_{0}^{3}}$$ 4

where δ_rad_ represents acoustic reradiation damping to the surrounding liquid with speed of sound *c*_L_, δ_*vis*_ accounts for viscous dissipation in a liquid with viscosity μ_L_ and density ρ, and δ_shell_ corresponds to damping due to shell viscosity κ_*s*_. Thermal damping due heat flux from the gas core to the surrounding liquid for these bubble sizes is comparable to viscous damping and for the purpose of simplification, the liquid viscosity μ_L_ was multiplied by a factor of 2.^4,6,52^

The energy loss of the insonifying acoustic wave due to damping is proportional to the acoustic scattering cross section Ω_*s*_, which is given by

$$\Omega_{s}=\frac{4\pi R_{0}^{2}}{{\left({\left(\omega_{0}/\omega \right)}^{2}-1\right)}^{2}+\delta_{\mathrm{tot}}^{2}}$$ 5

with δ_tot_ = δ_rad_ + δ_vis_ + δ_shell_. The attenuation coefficient can then be calculated using

$$\alpha_{\mathrm{model}}=-\frac{10}{\ln\;10}\underset{n}{\sum }\frac{\delta_{\mathrm{tot}}}{\delta_{\mathrm{rad}}}n\left(R_{n}\right)\Omega_{s}\left(R_{n}\right)d_{\mathrm{ac}}$$ 6

with *d*_ac_ the acoustical path length (see Figure 1C).

The root-mean-square (RMS) error between α_model_ and α_exp_:

$$\mathrm{RMS}=\sqrt{\underset{f_{T}}{\sum }{\left(\alpha_{\exp}\left(f_{T}\right)-\alpha_{\mathrm{model}}\left(f_{T}\right)\right)}^{2}}$$ 7

was minimized to find a best fit for the surface elasticity and surface viscosity for each attenuation curve at each ambient pressure *P*_amb_. Figure 1D shows two typical model fits (solid curves) to attenuation spectra measured at atmospheric pressure (dots) for two bubble suspensions with identical modal radii of 2.4 μm and polydispersity indices of 7% (standard deviation divided by the mean bubble size), but with different molar PA content. The obtained shell elasticity values were 0.5 N/m for the nondoped bubbles and 1.4 N/m for the 30 mol % PA-doped bubbles.

The surface tension curve σ­(*A*) was obtained by integration:

$$\sigma \left(A\right)=\int_{0}^{A}A^{-1}\chi \left(A\right)dA$$ 8

Once the interfacial tension reaches the surface tension of water, bubbles rapidly grow by diffusive gas influx as the surrounding liquid becomes oversaturated at reduced ambient pressures. This process differs between the acoustic setup and the IBIDI slide used for sizing because of the difference in bubble concentration. We therefore restrict the analysis to the regime where shell elasticity is nonzero. The obtained σ­(*A*) and κ_*s*_(*A*) curves were used as input in eqs 3 and 4, respectively, during the second run of the fitting procedure. Thus, the fitting procedure was repeated and for each run, σ­(*A*) and κ_*s*_(*A*) obtained at run *n* were used as input for run *n* + 1 until the σ­(*A*) curve converged (RMS error of run *n* + 1 was less than 10^–5^ different from run *n*).

## Results and Discussion

### Viscoelastic Properties at Atmospheric Pressure

Figure 2A presents the shell elasticity χ values measured at atmospheric pressure for 36 monodisperse microbubble suspensions with radii ranging from 1.5 to 4.5 μm. The palmitic acid (PA) concentration ranged from 0 to 80 mol % in 10 mol % increments, with at least 3 differently sized monodisperse microbubble suspensions measured at each concentration. The data in Figure 2A shows that the shell elasticity was independent of bubble size, which is consistent with our previous work.^4^ Therefore, the mean shell elasticity at each PA concentration (indicated by the horizontal lines in Figure 2A) was determined and plotted in Figure 2B. Note that, PA doping has a dramatic effect on shell elasticity, allowing control over shell elasticity across an order of magnitude—from 0.5 to 4.5 N/m—by increasing the PA content up to 50 mol %. Further increasing the PA concentration beyond 50 mol % led to a decrease in shell elasticity. Thus, doping the lipid shell with PA enables control over shell elasticity of monodisperse lipid-coated microbubbles reaching a maximum elasticity higher than previously reported in literature.

The shell viscosity κ_*s*_, plotted in Figure 2C, shows a clear dependence on bubble size. As frequently reported, shell viscosity follows an exponential relationship with bubble radius.^4,54,55^ Accordingly, the data in Figure 2C were fitted to the empirical exponential relation κ_*s*_ = *c*_1_ · *e* ^*c*_2_ *R*^ for each concentration, where *c*_2_ was fixed at 8.45 × 10^5^, and *c*_1_ was varied with PA concentration, as follows: 1.1854, 1.1854, 1.6018, 2.6541, 4.1274, 2.7406, 0.9127, and 0.3417 for PA concentrations of 0, 20, 30, 40, 50, 60, 70, and 80 mol %, respectively. The fitted curves are represented by the solid lines in Figure 2C. To visualize the relationship between shell viscosity and PA concentration, κ_*s*_ was calculated using the fitted curves for a bubble radius of 2.5 μm, and the resulting trend is shown in Figure 2D. Interestingly, the data reveal that shell viscosity and shell elasticity are not decoupled as assumed in all viscoelastic bubble shell models; rather, they follow a similar trend, both reaching a maximum at 50 mol % PA. This result suggests that not only shell elasticity, but also shell damping, originates from the lateral intermolecular forces within the lipid monolayer shell.^56^

Prior work on Langmuir monolayers of DPPC:PA mixtures provides two possible explanations for the observed effects. First, grazing incidence X-ray diffraction (GIXD) studies of Langmuir trough monolayers indicated that the tilt angle decreased from ∼20° for pure DPPC to ∼5° for DPPC:PA = 1:1 mixtures, providing closer packing of the hydrocarbon chains and increased coherence length of the crystalline domains.^57^ In the shell of a microbubble, improved lipid-chain packing induced by the PA would increase the cohesive intermolecular van der Waals forces between the acyl chains, thereby explaining the increased elasticity and viscosity with increasing PA content. Alternatively, vibrational sum frequency generation (VSFG) spectra on Langmuir monolayers of DPPC:PA mixtures indicated hydrogen bond formation between the DPPC phosphate group and the PA hydroxyl group, evidenced by a red shift in the phosphate symmetric stretch.^45^ The hydrogen bond would increase cohesion within the lipid shell, thereby explaining the increased elasticity and viscosity with increasing PA content.

The drop in lipid shell elasticity and viscosity for PA concentrations above 50 mol % is also consistent with prior work on Langmuir trough monolayers.^45,57^ The shape of the surface pressure–area isotherms changes for PA concentrations above 50 mol %, indicating that the excess PA demixes from the DPPC domains to form coexisting PA-rich domains. This change in microstructure from DPPC:PA domains to DPPC:PA domains coexisting with PA domains apparently softens the microbubble shells, which is consistent with our observation in the next section that such microbubbles are unstable to surface compression.

### Dilatational Surface Elasticity and Viscosity Curves

Figure 3 shows dilatational shell elasticity (first column), surface tension (second column), and shell viscosity (third column) plotted as a function of normalized bubble surface area *A*/*A*_0_, with *A*_0_ the bubble surface area at atmospheric pressure (4π*R*_0_^2^). Data are shown for four molar palmitic acid concentrations: 0, 20, 30, and 50%. To aid comparison, the first row shows data for both 0 mol % and 20 mol % PA, the second row for 20 and 30% PA, and the third for 0 and 50% PA. The modal radii of the four bubble suspensions were 2.4 μm for 0% and 30 mol % PA, 3.2 μm for 20 mol % PA, and 3.1 μm for 50 mol % PA. The PDI of all bubble suspension was 7%.

For 0 mol % PA (gray data points in Figure 3A), the elasticity curve shows that when these microbubbles are compressed, the surface elasticity quickly drops from its maximum value of 0.55 N/m to zero. During expansion, the surface elasticity first gradually drops to 0.4 N/m, and then more rapidly to zero. The corresponding surface tension curve (Figure 3B) varies between 0 N/m and 72 mN/m with a decreasing slope as the monolayer dilates. The initial surface tension (σ­(*A*/*A*_0_ = 1)) is very close to zero such that the Laplace pressure (2σ/*R*_0_) is near zero, stabilizing the bubble against dissolution. The low initial surface tension causes even small radial strain amplitudes to average out the surface area–dependent shell elasticity, thereby reducing the effective shell elasticity with increasing radial strain. Since radial strain depends on bubble size through resonance, this may explain why other studies have reported a bubble size–dependent shell elasticity.^55,58^

The dilatational surface elasticity and surface tension curves for 0 mol % PA, i.e., for DSPC-DPPE-PEG5000 (9:1 molar ratio) coated microbubbles (Figure 3) closely resemble those of DPPC-DPPE-PEG5000 (9:1 molar ratio) coated microbubbles previously obtained by Segers et al.^4^ Thus, the increased acyl chain length of DSPC (di-C18:0) compared to DPPC (di-C16:0) does not result in a stiffer monolayer shell when mixed with 10 mol % DPPE-PEG5000. This finding contrasts with the results of Lum et al.,^38^ who observed an increasing trend in shell elasticity with longer acyl chains. Their use of a longer C18 phospholipid anchor and a shorter PEG chain (DSPE-PEG2000) may account for the discrepancy.

The dilatational shell viscosity measured for 0 mol % PA is plotted in Figure 3C as gray data points. It appears that there are the following regimes: *A*/*A*_0_ < 1: shell viscosity steeply drops with compression, following the trend with elasticity, presumably due to buckling. One < *A*/*A*_0_ < 1.1: shell viscosity decreases monotonically with expansion, following the trend with elasticity. This is presumably the “elastic regime” where the shell is a continuous monolayer that is neither buckled nor ruptured, completely coating the gas core. 1.1 < *A*/*A*_0_ < 1.15: shell viscosity increases with further expansion, no longer following the trend with elasticity, which may indicate rupture. 1.15 < *A*/*A*_0_ < 1.2: shell viscosity peaks and then decreases with further expansion. As demonstrated by Doinikov et al.,^59^ a nonlinear shell viscosity that varies with bubble surface area can boost the nonlinear microbubble response, underscoring the need for further research into the contributions of shell elasticity and viscosity to the nonlinear bubble response. A simultaneous increase in shell viscosity and decrease in elasticity upon shell dilatation has been previously reported by Thomas et al.,^60^ who identified it as “a hallmark of interfacial melting and spreading”. The idea is that liquids exhibit higher viscosity than solids, which tend to rupture rather than expand under forced surface dilatation. Despite these insights, the quantitative relationship between elastic and viscous effects remains unclear for now, as well as the increased shell damping for the highly compressed bubble shell (*A*/*A*_0_ < 1.05).

The effect of doping the shell with 20 mol % PA on the dilatational surface elasticity (blue data points in Figure 3A) is an increase in maximum shell elasticity from 0.55 N/m in the case of 0 mol % to 0.8 N/m for 20 mol %. This maximum surface elasticity of 0.8 N/m is reached only in a small region close to *A*/*A*_0_ = 1. Note that the curves for 0 and 20 mol % PA are almost identical outside this region. As noted above, prior work on Langmuir monolayers of DPPC:PA mixtures indicated increased acyl chain ordering^57^ and hydrogen bond formation between the DPPC phosphate group and the PA hydroxyl group.^45^ The short-range nature of these interactions may explain the small region of dilatation over which the increased elasticity is observed. The results indicate that the condensing effect of PA due to hydrogen bond formation and reduction in the acyl chain tilt angle, is lost once the bubble area is increased by only 1%. Due to the increased shell elasticity at the small 1% region around *A*/*A*_0_ = 1, the corresponding surface tension curve (blue data points in Figure 3B) shows only a minor difference in slope around *A*/*A*_0_ = 1, compared to that for 0 mol % PA. The shell viscosity curves of the 0 and 20 mol % PA bubbles (Figure 3C) are very similar in shape, with the shell viscosity of the 20 mol % PA bubbles increasing to higher values during bubble compression and expansion.

Figure 3D shows that for 30 mol % PA, shell elasticity increases to a maximum value of 1.8 N/m, similar to the increase observed in Figure 2B. Again, the increased shell elasticity is centered around a narrow region around *A*/*A*_0_ = 1. Outside of this high surface elasticity region the difference with the 0 and 20 mol % PA bubbles is small. Consequently, the surface tension curve of the 30 mol % PA bubbles (orange data points in Figure 3E) is very similar to that of the 0 and 20 mol % PA bubbles except for its higher slope around *A*/*A*_0_ = 1. Notably, the shell viscosity curves for the 20 and 30 mol % PA bubbles are very similar.

The dilatational surface elasticity of the 50 mol % PA bubbles (red data points in Figure 3G) shows an increased maximum surface elasticity of 4.5 N/m, again at a narrow region at *A*/*A*_0_ = 1. The resulting surface tension curve in Figure 3H is the steepest curve that could be obtained. Note that is has two distinct regions, one with a high slope corresponding to the high elasticity region, and one with a lower slope corresponding to the lower elasticity region. The dilatational viscosity curve in Figure 3I follows the same trend as before, with a maximum κ_*s*_ for the compressed bubble shell (*A*/*A*_0_ ≈ 1) and for the ruptured bubble shell (*A*/*A*_0_ ≈ 1.1).

Shell elasticity and shell viscosity curves for 60 mol % PA and higher could not be obtained, due to bubble instability. As the ambient pressure increased above atmospheric pressure, the frequency of maximum attenuation dropped to a low frequency, indicating buckling and a loss of shell elasticity—similar to the observations at lower PA concentrations. However, once buckled, the microbubbles regained their surface elasticity, and their resonance frequency continued to switch between higher and lower values, i.e., the shell continued to switch between the elastic and buckled state. Regaining surface elasticity under compression is only possible with an increase in the surface tension, as the tensionless buckled state has zero surface elasticity—implying a loss of shell material, which is indeed linked to shell buckling.^29,61^ A similar instability has been observed in Langmuir trough experiments with PA and DPPC monolayers, where for 75 mol % PA monolayer collapse occurs when a surface pressure of 40 mN/m is exceeded,^45,57^ while a surface pressure equal to the surface tension of water (72 mN/m) is necessary to stabilize the microbubble against Laplace pressure-driven dissolution. A follow-up study investigating how both size stability and acoustic stability depend on PA content would be highly relevant.

In the present work, the time scale of microbubble surface area variations was limited by the experimental setup controlling the ambient pressure, and was restricted to seconds. Obtaining dilatational surface elasticity and viscosity curves at shorter time scales, down to the μs range of ultrasound-driven bubble oscillations, is of great interest. While this remains speculative, we expect the resulting surface tension curves to be similar, as our current approach produces curves comparable to those of the Marmottant model, which has been shown to accurately capture nonlinear oscillation phenomena of phospholipid-coated microbubbles. Furthermore, recent stress–strain analysis of single ultrasound-driven microbubbles using ultrasound-induced radial strain have yielded very similar surface tension curves, supporting this expectation.^62^

### Effect of PA Doping on Acoustic Bubble Response

The surface tension curve for 0 mol % PA increases nearly linearly from zero to 72 mN/m, approximately following the ad-hoc assumption made in the Marmottant model.^3^ However, as we now show, doping the shell with PA can result in a very different surface tension curve, which is not only steeper (higher shell elasticity), but also has a different shape with a high and lower slope region. The influence of this very different surface tension curve on the acoustic response of a microbubble is investigated by numerically solving eq 2 using the dilatational surface tension curves obtained at 0 and 50 mol % PA. The σ­(*A*) data points as shown in Figure 3G were used in a look-up table were surface tension at each time step in the numerical ODE solver was found from a linear interpolation of the measured σ­(*A*) curves. Outside of the range of the σ­(*A*) data points the surface tension was set to zero at small radii and to that of water, 0.072 N/m, for large radii. The other input parameters were the density of water ρ = 1000 kg m^–3^, the viscosity of water μ = 1 mPa s, the speed of sound in water c = 1500 ms^–1^, the polytropic exponent κ = 1.07 of C_4_F_10_ gas, the ambient pressure *P*_0_ = 100 kPa, and an initial microbubble radius *R*_*i*_ of 2.5 μm. The driving pulse had a pressure amplitude of 75 kPa and a length of 16-cycles tapered over 3 cycles at the start and end of the pulse. The frequency of the driving pulse *f*_*T*_ was varied from 0.5 to 8 MHz in steps of 100 kHz. The obtained radius-time curves were used to obtain the scattered pressure calculated at a distance *r* = 2.54 cm, using *P*_*s*_(*t*) = ρ_*L*_ *r* ^–1^(*R* ^2^ *R̈* + 2*RṘ* ^2^).^63^

Figure 4A,B present the scattered power maps for a bubble without PA and one doped with 50 mol % PA, respectively. Figure 4C,D show the scattered power as a function of the ultrasound driving frequency along the fundamental (*y* = *x* in Figure 4A,B), second harmonic (*y* = 2*x*), and subharmonic (*y* = 0.5*x*). The fundamental and second harmonic responses are approximately 5 dB stronger for the non-PA-doped bubbles. In contrast, the subharmonic scattered power of the PA-doped bubble is enhanced by 20 dB compared to the non-PA-doped bubble. These results show that, under the applied acoustic conditions, the increased shell elasticity boosts the subharmonic response, as predicted before.^6,64^ These simulations thereby demonstrate that the controlled shell elasticity offered by this work allows microbubbles to be tuned in nonlinear response, which may greatly aid in increasing the sensitivity of the subharmonic response of microbubbles designed for noninvasive pressure sensing.^11,13^

## Conclusions

This study demonstrates that the shell elasticity of DSPC and DPPE-PEG5000 coated bubbles can be controlled over 1 order of magnitude, from 0.5 N/m to 4.5 N/m by doping the shell with palmitic acid. The maximum shell elasticity of 4.5 N/m is reached at a concentration of 50 mol % PA. Dilatational surface elasticity and corresponding surface tension curves of PA-doped bubbles reveal that the increased shell elasticity is confined to a narrow region around the equilibrium bubble surface area. Upon expansion of just 1–2*%* in bubble surface area, surface elasticity rapidly drops to levels observed in non-PA-doped bubbles. The results also show that shell viscosity varies with bubble surface area, which may further boost nonlinear bubble dynamics. The increased shell elasticity and corresponding steeper surface tension curve near the buckling point result in a strong subharmonic acoustic response of the bubble, enabling the design of bubbles with tailored and increased nonlinear acoustic properties. This may be particularly beneficial for nonlinear imaging techniques and subharmonic-aided pressure sensing.
